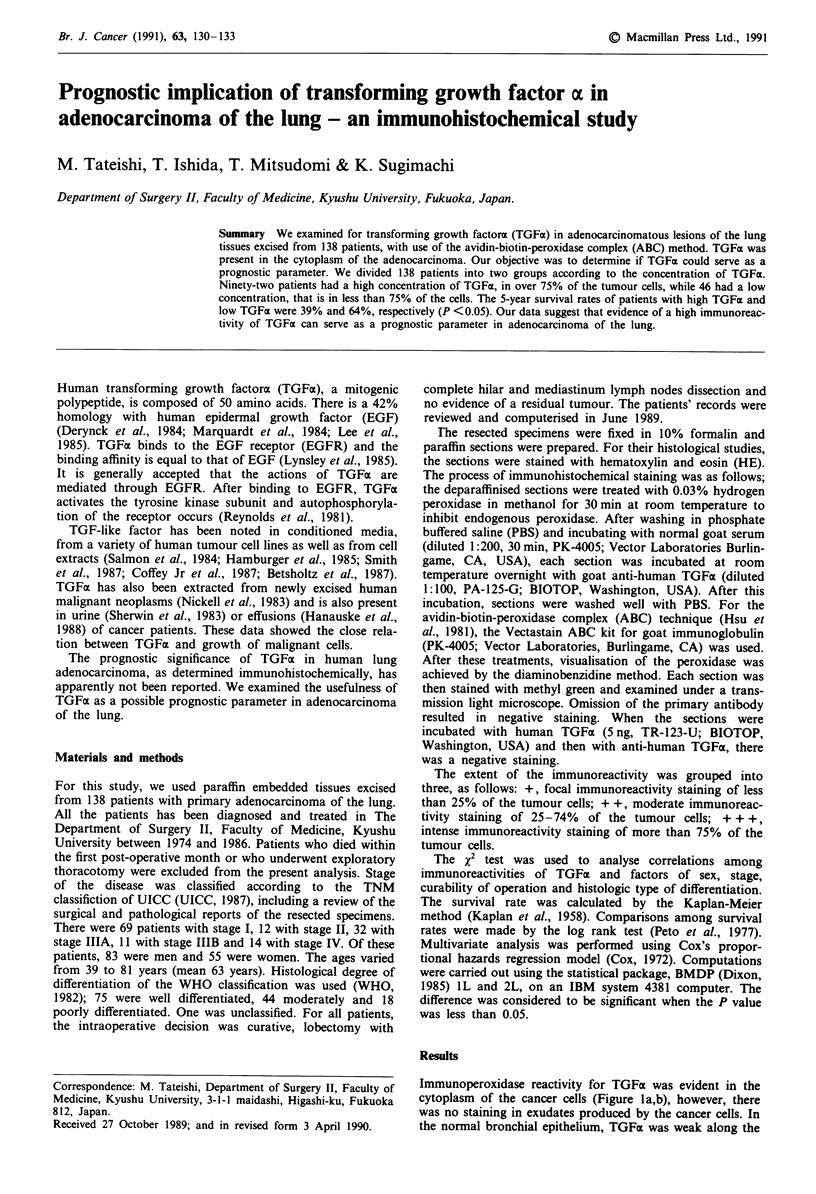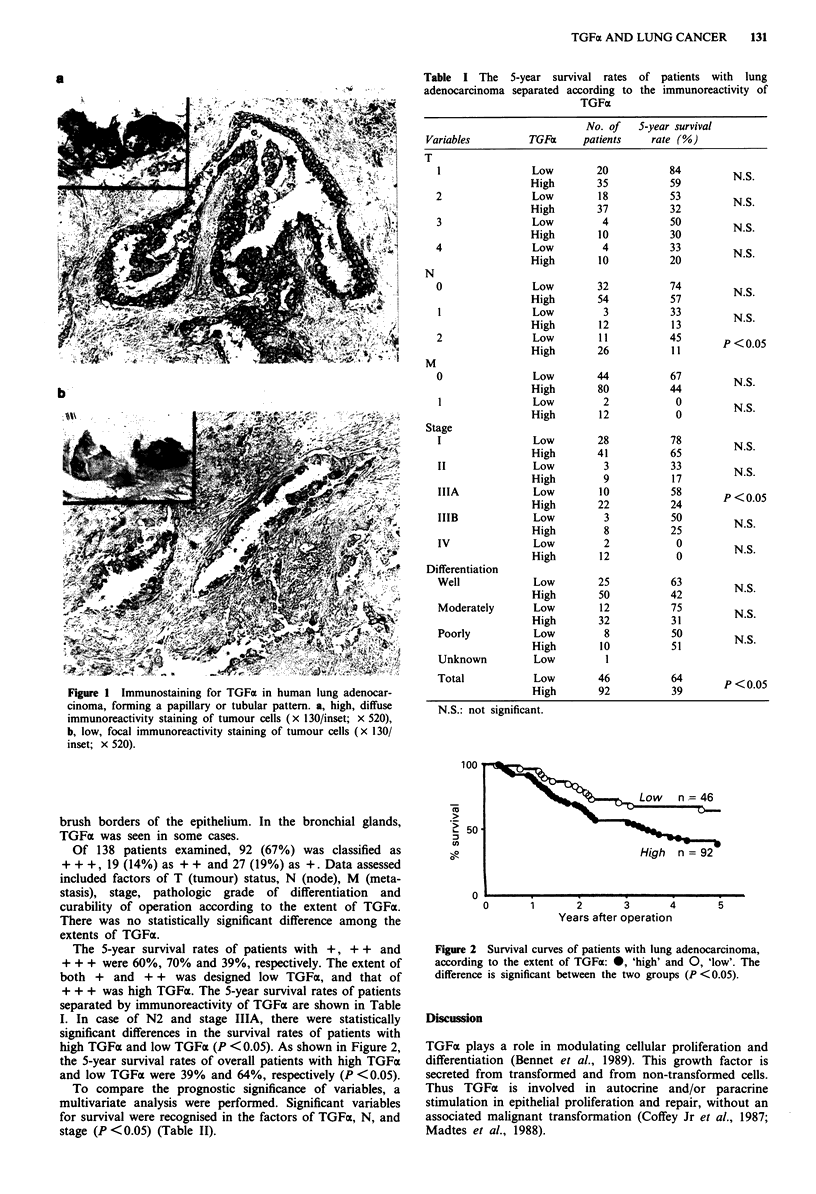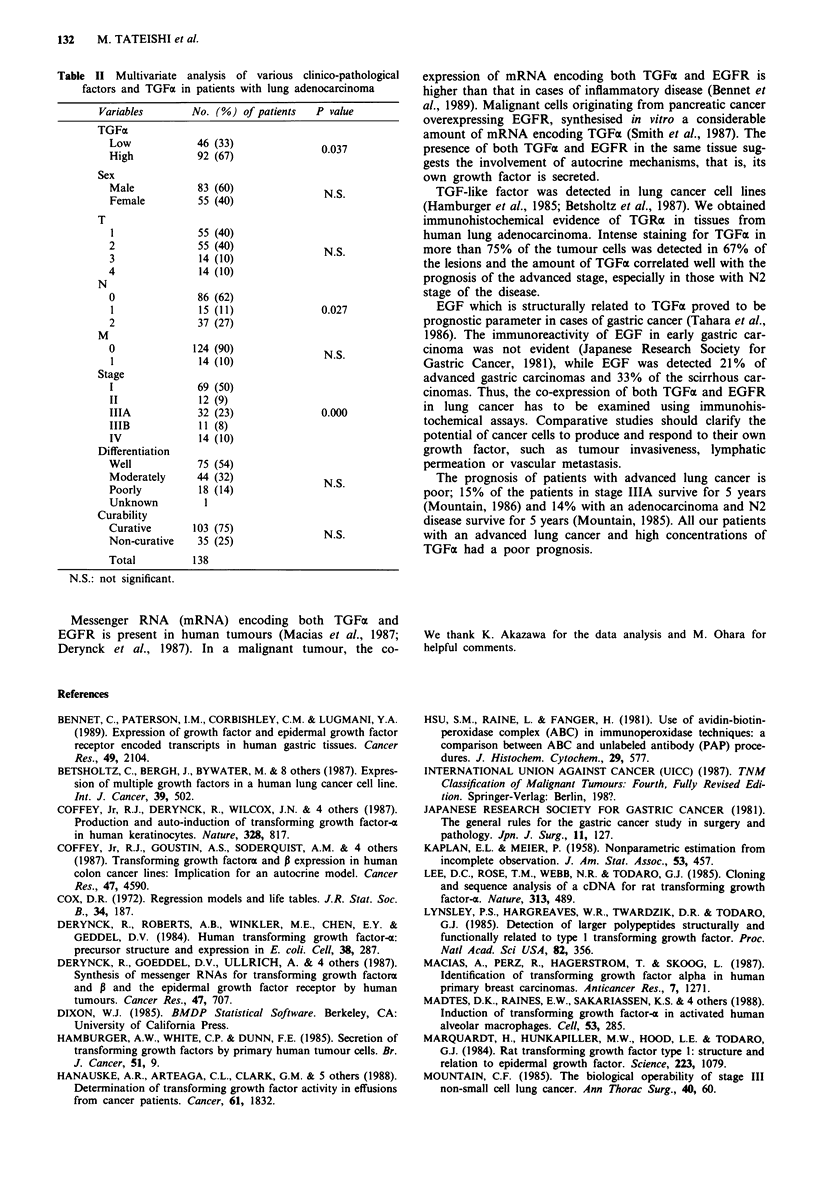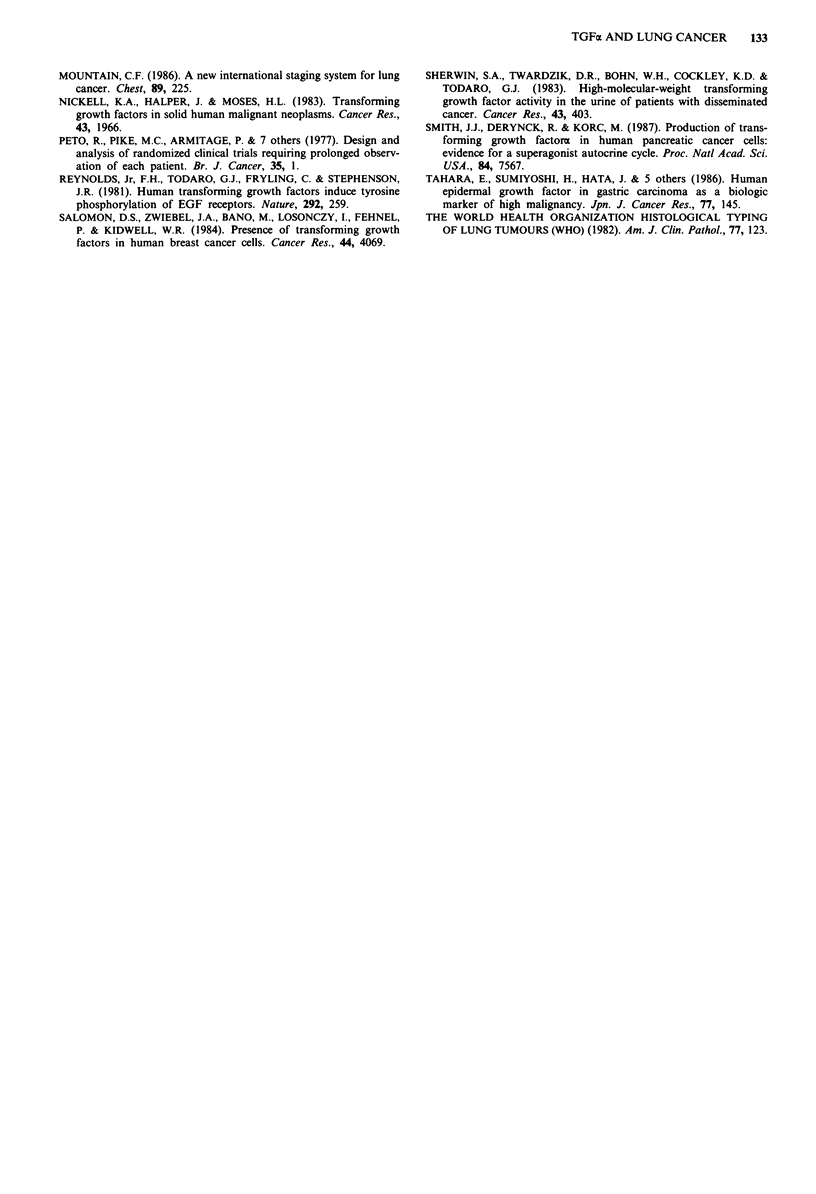# Prognostic implication of transforming growth factor alpha in adenocarcinoma of the lung--an immunohistochemical study.

**DOI:** 10.1038/bjc.1991.26

**Published:** 1991-01

**Authors:** M. Tateishi, T. Ishida, T. Mitsudomi, K. Sugimachi

**Affiliations:** Department of Surgery II, Faculty of Medicine, Kyushu University, Fukuoka, Japan.

## Abstract

**Images:**


Br. J. Cancer (1991), 63, 130–133 © Macmillan Press Ltd., 1991

### Prognostic implication of transforming growth factor α in adenocarcinoma of the lung – an immunohistochemical study

M. Tateishi, T. Ishida, T. Mitsudomi & K. Sugimachi

*Department of Surgery II, Faculty of Medicine, Kyushu University, Fukuoka, Japan.*

**Summary** We examined for transforming growth factorα (TGFα) in adenocarcinomatous lesions of the lung tissues excised from 138 patients, with use of the avidin-biotin-peroxidase complex (ABC) method. TGFα was present in the cytoplasm of the adenocarcinoma. Our objective was to determine if TGFα could serve as a prognostic parameter. We divided 138 patients into two groups according to the concentration of TGFα. Ninety-two patients had a high concentration of TGFα, in over 75% of the tumour cells, while 46 had a low concentration, that is in less than 75% of the cells. The 5-year survival rates of patients with high TGFα and low TGFα were 39% and 64%, respectively ($P < 0.05$). Our data suggest that evidence of a high immunoreactivity of TGFα can serve as a prognostic parameter in adenocarcinoma of the lung.

Human transforming growth factorα (TGFα), a mitogenic polypeptide, is composed of 50 amino acids. There is a 42% homology with human epidermal growth factor (EGF) (Derynck *et al.*, 1984; Marquardt *et al.*, 1984; Lee *et al.*, 1985). TGFα binds to the EGF receptor (EGFR) and the binding affinity is equal to that of EGF (Lynsley *et al.*, 1985). It is generally accepted that the actions of TGFα are mediated through EGFR. After binding to EGFR, TGFα activates the tyrosine kinase subunit and autophosphorylation of the receptor occurs (Reynolds *et al.*, 1981).

TGF-like factor has been noted in conditioned media, from a variety of human tumour cell lines as well as from cell extracts (Salmon *et al.*, 1984; Hamburger *et al.*, 1985; Smith *et al.*, 1987; Coffey Jr *et al.*, 1987; Betsholtz *et al.*, 1987). TGFα has also been extracted from newly excised human malignant neoplasms (Nickell *et al.*, 1983) and is also present in urine (Sherwin *et al.*, 1983) or effusions (Hanauske *et al.*, 1988) of cancer patients. These data showed the close relation between TGFα and growth of malignant cells.

The prognostic significance of TGFα in human lung adenocarcinoma, as determined immunohistochemically, has apparently not been reported. We examined the usefulness of TGFα as a possible prognostic parameter in adenocarcinoma of the lung.

### Materials and methods

For this study, we used paraffin embedded tissues excised from 138 patients with primary adenocarcinoma of the lung. All the patients has been diagnosed and treated in The Department of Surgery II, Faculty of Medicine, Kyushu University between 1974 and 1986. Patients who died within the first post-operative month or who underwent exploratory thoracotomy were excluded from the present analysis. Stage of the disease was classified according to the TNM classifiction of UICC (UICC, 1987), including a review of the surgical and pathological reports of the resected specimens. There were 69 patients with stage I, 12 with stage II, 32 with stage IIIA, 11 with stage IIIB and 14 with stage IV. Of these patients, 83 were men and 55 were women. The ages varied from 39 to 81 years (mean 63 years). Histological degree of differentiation of the WHO classification was used (WHO, 1982); 75 were well differentiated, 44 moderately and 18 poorly differentiated. One was unclassified. For all patients, the intraoperative decision was curative, lobectomy with complete hilar and mediastinum lymph nodes dissection and no evidence of a residual tumour. The patients' records were reviewed and computerised in June 1989.

The resected specimens were fixed in 10% formalin and paraffin sections were prepared. For their histological studies, the sections were stained with hematoxylin and eosin (HE). The process of immunohistochemical staining was as follows; the deparaffinised sections were treated with 0.03% hydrogen peroxidase in methanol for 30 min at room temperature to inhibit endogenous peroxidase. After washing in phosphate buffered saline (PBS) and incubating with normal goat serum (diluted 1:200, 30 min, PK-4005; Vector Laboratories Burlingame, CA, USA), each section was incubated at room temperature overnight with goat anti-human TGFα (diluted 1:100, PA-125-G; BIOTOP, Washington, USA). After this incubation, sections were washed well with PBS. For the avidin-biotin-peroxidase complex (ABC) technique (Hsu *et al.*, 1981), the Vectastain ABC kit for goat immunoglobulin (PK-4005; Vector Laboratories, Burlingame, CA) was used. After these treatments, visualisation of the peroxidase was achieved by the diaminobenzidine method. Each section was then stained with methyl green and examined under a transmission light microscope. Omission of the primary antibody resulted in negative staining. When the sections were incubated with human TGFα (5 ng, TR-123-U; BIOTOP, Washington, USA) and then with anti-human TGFα, there was a negative staining.

The extent of the immunoreactivity was grouped into three, as follows: +, focal immunoreactivity staining of less than 25% of the tumour cells; + +, moderate immunoreactivity staining of 25–74% of the tumour cells; + + +, intense immunoreactivity staining of more than 75% of the tumour cells.

The $\chi^2$ test was used to analyse correlations among immunoreactivities of TGFα and factors of sex, stage, curability of operation and histologic type of differentiation. The survival rate was calculated by the Kaplan-Meier method (Kaplan *et al.*, 1958). Comparisons among survival rates were made by the log rank test (Peto *et al.*, 1977). Multivariate analysis was performed using Cox's proportional hazards regression model (Cox, 1972). Computations were carried out using the statistical package, BMDP (Dixon, 1985) 1L and 2L, on an IBM system 4381 computer. The difference was considered to be significant when the $P$ value was less than 0.05.

### Results

Immunoperoxidase reactivity for TGFα was evident in the cytoplasm of the cancer cells (Figure 1a,b), however, there was no staining in exudates produced by the cancer cells. In the normal bronchial epithelium, TGFα was weak along the

Correspondence: M. Tateishi, Department of Surgery II, Faculty of Medicine, Kyushu University, 3-1-1 maidashi, Higashi-ku, Fukuoka 812, Japan.
Received 27 October 1989; and in revised form 3 April 1990.

**Figure 1** Immunostaining for TGFα in human lung adenocarcinoma, forming a papillary or tubular pattern. **a**, high, diffuse immunoreactivity staining of tumour cells (× 130/inset; × 520), **b**, low, focal immunoreactivity staining of tumour cells (× 130/inset; × 520).

brush borders of the epithelium. In the bronchial glands, TGFα was seen in some cases.

Of 138 patients examined, 92 (67%) was classified as + + +, 19 (14%) as + + and 27 (19%) as +. Data assessed included factors of T (tumour) status, N (node), M (metastasis), stage, pathologic grade of differentiation and curability of operation according to the extent of TGFα. There was no statistically significant difference among the extents of TGFα.

The 5-year survival rates of patients with +, + + and + + + were 60%, 70% and 39%, respectively. The extent of both + and + + was designed low TGFα, and that of + + + was high TGFα. The 5-year survival rates of patients separated by immunoreactivity of TGFα are shown in Table I. In case of N2 and stage IIIA, there were statistically significant differences in the survival rates of patients with high TGFα and low TGFα ($P<0.05$). As shown in Figure 2, the 5-year survival rates of overall patients with high TGFα and low TGFα were 39% and 64%, respectively ($P<0.05$).

To compare the prognostic significance of variables, a multivariate analysis were performed. Significant variables for survival were recognised in the factors of TGFα, N, and stage ($P<0.05$) (Table II).

**Table I** The 5-year survival rates of patients with lung adenocarcinoma separated according to the immunoreactivity of TGFα

| *Variables* | *TGFα* | *No. of patients* | *5-year survival rate (%)* | |
|---|---|---|---|---|
| T | | | | |
| 1 | Low | 20 | 84 | N.S. |
| | High | 35 | 59 | |
| 2 | Low | 18 | 53 | N.S. |
| | High | 37 | 32 | |
| 3 | Low | 4 | 50 | N.S. |
| | High | 10 | 30 | |
| 4 | Low | 4 | 33 | N.S. |
| | High | 10 | 20 | |
| N | | | | |
| 0 | Low | 32 | 74 | N.S. |
| | High | 54 | 57 | |
| 1 | Low | 3 | 33 | N.S. |
| | High | 12 | 13 | |
| 2 | Low | 11 | 45 | $P<0.05$ |
| | High | 26 | 11 | |
| M | | | | |
| 0 | Low | 44 | 67 | N.S. |
| | High | 80 | 44 | |
| 1 | Low | 2 | 0 | N.S. |
| | High | 12 | 0 | |
| Stage | | | | |
| I | Low | 28 | 78 | N.S. |
| | High | 41 | 65 | |
| II | Low | 3 | 33 | N.S. |
| | High | 9 | 17 | |
| IIIA | Low | 10 | 58 | $P<0.05$ |
| | High | 22 | 24 | |
| IIIB | Low | 3 | 50 | N.S. |
| | High | 8 | 25 | |
| IV | Low | 2 | 0 | N.S. |
| | High | 12 | 0 | |
| Differentiation | | | | |
| Well | Low | 25 | 63 | N.S. |
| | High | 50 | 42 | |
| Moderately | Low | 12 | 75 | N.S. |
| | High | 32 | 31 | |
| Poorly | Low | 8 | 50 | N.S. |
| | High | 10 | 51 | |
| Unknown | Low | 1 | | |
| Total | Low | 46 | 64 | $P<0.05$ |
| | High | 92 | 39 | |

N.S.: not significant.

**Figure 2** Survival curves of patients with lung adenocarcinoma, according to the extent of TGFα: ●, 'high' and O, 'low'. The difference is significant between the two groups ($P<0.05$).

### Discussion

TGFα plays a role in modulating cellular proliferation and differentiation (Bennet *et al.*, 1989). This growth factor is secreted from transformed and from non-transformed cells. Thus TGFα is involved in autocrine and/or paracrine stimulation in epithelial proliferation and repair, without an associated malignant transformation (Coffey Jr *et al.*, 1987; Madtes *et al.*, 1988).

**Table II** Multivariate analysis of various clinico-pathological factors and TGFα in patients with lung adenocarcinoma

| *Variables* | *No. (%) of patients* | *P value* |
|---|---|---|
| TGFα | | |
| Low | 46 (33) | 0.037 |
| High | 92 (67) | |
| Sex | | |
| Male | 83 (60) | N.S. |
| Female | 55 (40) | |
| T | | |
| 1 | 55 (40) | |
| 2 | 55 (40) | N.S. |
| 3 | 14 (10) | |
| 4 | 14 (10) | |
| N | | |
| 0 | 86 (62) | |
| 1 | 15 (11) | 0.027 |
| 2 | 37 (27) | |
| M | | |
| 0 | 124 (90) | N.S. |
| 1 | 14 (10) | |
| Stage | | |
| I | 69 (50) | |
| II | 12 (9) | |
| IIIA | 32 (23) | 0.000 |
| IIIB | 11 (8) | |
| IV | 14 (10) | |
| Differentiation | | |
| Well | 75 (54) | |
| Moderately | 44 (32) | N.S. |
| Poorly | 18 (14) | |
| Unknown | 1 | |
| Curability | | |
| Curative | 103 (75) | N.S. |
| Non-curative | 35 (25) | |
| Total | 138 | |

N.S.: not significant.

Messenger RNA (mRNA) encoding both TGFα and EGFR is present in human tumours (Macias *et al.*, 1987; Derynck *et al.*, 1987). In a malignant tumour, the co-expression of mRNA encoding both TGFα and EGFR is higher than that in cases of inflammatory disease (Bennet *et al.*, 1989). Malignant cells originating from pancreatic cancer overexpressing EGFR, synthesised *in vitro* a considerable amount of mRNA encoding TGFα (Smith *et al.*, 1987). The presence of both TGFα and EGFR in the same tissue suggests the involvement of autocrine mechanisms, that is, its own growth factor is secreted.

TGF-like factor was detected in lung cancer cell lines (Hamburger *et al.*, 1985; Betsholtz *et al.*, 1987). We obtained immunohistochemical evidence of TGRα in tissues from human lung adenocarcinoma. Intense staining for TGFα in more than 75% of the tumour cells was detected in 67% of the lesions and the amount of TGFα correlated well with the prognosis of the advanced stage, especially in those with N2 stage of the disease.

EGF which is structurally related to TGFα proved to be prognostic parameter in cases of gastric cancer (Tahara *et al.*, 1986). The immunoreactivity of EGF in early gastric carcinoma was not evident (Japanese Research Society for Gastric Cancer, 1981), while EGF was detected 21% of advanced gastric carcinomas and 33% of the scirrhous carcinomas. Thus, the co-expression of both TGFα and EGFR in lung cancer has to be examined using immunohistochemical assays. Comparative studies should clarify the potential of cancer cells to produce and respond to their own growth factor, such as tumour invasiveness, lymphatic permeation or vascular metastasis.

The prognosis of patients with advanced lung cancer is poor; 15% of the patients in stage IIIA survive for 5 years (Mountain, 1986) and 14% with an adenocarcinoma and N2 disease survive for 5 years (Mountain, 1985). All our patients with an advanced lung cancer and high concentrations of TGFα had a poor prognosis.

We thank K. Akazawa for the data analysis and M. Ohara for helpful comments.

### References

BENNET, C., PATERSON, I.M., CORBISHLEY, C.M. & LUGMANI, Y.A. (1989). Expression of growth factor and epidermal growth factor receptor encoded transcripts in human gastric tissues. *Cancer Res.*, **49**, 2104.

BETSHOLTZ, C., BERGH, J., BYWATER, M. & 8 others (1987). Expression of multiple growth factors in a human lung cancer cell line. *Int. J. Cancer*, **39**, 502.

COFFEY, Jr, R.J., DERYNCK, R., WILCOX, J.N. & 4 others (1987). Production and auto-induction of transforming growth factor-α in human keratinocytes. *Nature*, **328**, 817.

COFFEY, Jr, R.J., GOUSTIN, A.S., SODERQUIST, A.M. & 4 others (1987). Transforming growth factorα and β expression in human colon cancer lines: Implication for an autocrine model. *Cancer Res.*, **47**, 4590.

COX, D.R. (1972). Regression models and life tables. *J.R. Stat. Soc. B.*, **34**, 187.

DERYNCK, R., ROBERTS, A.B., WINKLER, M.E., CHEN, E.Y. & GEDDEL, D.V. (1984). Human transforming growth factor-α: precursor structure and expression in *E. coli. Cell*, **38**, 287.

DERYNCK, R., GOEDDEL, D.V., ULLRICH, A. & 4 others (1987). Synthesis of messenger RNAs for transforming growth factorα and β and the epidermal growth factor receptor by human tumours. *Cancer Res.*, **47**, 707.

DIXON, W.J. (1985). *BMDP Statistical Software*. Berkeley, CA: University of California Press.

HAMBURGER, A.W., WHITE, C.P. & DUNN, F.E. (1985). Secretion of transforming growth factors by primary human tumour cells. *Br. J. Cancer*, **51**, 9.

HANAUSKE, A.R., ARTEAGA, C.L., CLARK, G.M. & 5 others (1988). Determination of transforming growth factor activity in effusions from cancer patients. *Cancer*, **61**, 1832.

HSU, S.M., RAINE, L. & FANGER, H. (1981). Use of avidin-biotin-peroxidase complex (ABC) in immunoperoxidase techniques: a comparison between ABC and unlabeled antibody (PAP) procedures. *J. Histochem. Cytochem.*, **29**, 577.

INTERNATIONAL UNION AGAINST CANCER (UICC) (1987). *TNM Classification of Malignant Tumours: Fourth, Fully Revised Edition*. Springer-Verlag: Berlin, 198?.

JAPANESE RESEARCH SOCIETY FOR GASTRIC CANCER (1981). The general rules for the gastric cancer study in surgery and pathology. *Jpn. J. Surg.*, **11**, 127.

KAPLAN, E.L. & MEIER, P. (1958). Nonparametric estimation from incomplete observation. *J. Am. Stat. Assoc.*, **53**, 457.

LEE, D.C., ROSE, T.M., WEBB, N.R. & TODARO, G.J. (1985). Cloning and sequence analysis of a cDNA for rat transforming growth factor-α. *Nature*, **313**, 489.

LYNSLEY, P.S., HARGREAVES, W.R., TWARDZIK, D.R. & TODARO, G.J. (1985). Detection of larger polypeptides structurally and functionally related to type 1 transforming growth factor. *Proc. Natl Acad. Sci USA*, **82**, 356.

MACIAS, A., PERZ, R., HAGERSTROM, T. & SKOOG, L. (1987). Identification of transforming growth factor alpha in human primary breast carcinomas. *Anticancer Res.*, **7**, 1271.

MADTES, D.K., RAINES, E.W., SAKARIASSEN, K.S. & 4 others (1988). Induction of transforming growth factor-α in activated human alveolar macrophages. *Cell*, **53**, 285.

MARQUARDT, H., HUNKAPILLER, M.W., HOOD, L.E. & TODARO, G.J. (1984). Rat transforming growth factor type 1: structure and relation to epidermal growth factor. *Science*, **223**, 1079.

MOUNTAIN, C.F. (1985). The biological operability of stage III non-small cell lung cancer. *Ann Thorac Surg.*, **40**, 60.

MOUNTAIN, C.F. (1986). A new international staging system for lung cancer. *Chest*, **89**, 225.

NICKELL, K.A., HALPER, J. & MOSES, H.L. (1983). Transforming growth factors in solid human malignant neoplasms. *Cancer Res.*, **43**, 1966.

PETO, R., PIKE, M.C., ARMITAGE, P. & 7 others (1977). Design and analysis of randomized clinical trials requiring prolonged observation of each patient. *Br. J. Cancer*, **35**, 1.

REYNOLDS, Jr, F.H., TODARO, G.J., FRYLING, C. & STEPHENSON, J.R. (1981). Human transforming growth factors induce tyrosine phosphorylation of EGF receptors. *Nature*, **292**, 259.

SALOMON, D.S., ZWIEBEL, J.A., BANO, M., LOSONCZY, I., FEHNEL, P. & KIDWELL, W.R. (1984). Presence of transforming growth factors in human breast cancer cells. *Cancer Res.*, **44**, 4069.

SHERWIN, S.A., TWARDZIK, D.R., BOHN, W.H., COCKLEY, K.D. & TODARO, G.J. (1983). High-molecular-weight transforming growth factor activity in the urine of patients with disseminated cancer. *Cancer Res.*, **43**, 403.

SMITH, J.J., DERYNCK, R. & KORC, M. (1987). Production of transforming growth factorα in human pancreatic cancer cells: evidence for a superagonist autocrine cycle. *Proc. Natl Acad. Sci. USA.*, **84**, 7567.

TAHARA, E., SUMIYOSHI, H., HATA, J. & 5 others (1986). Human epidermal growth factor in gastric carcinoma as a biologic marker of high malignancy. *Jpn. J. Cancer Res.*, **77**, 145.

THE WORLD HEALTH ORGANIZATION HISTOLOGICAL TYPING OF LUNG TUMOURS (WHO) (1982). *Am. J. Clin. Pathol.*, **77**, 123.